# Fibrinogen Concentrations in Liquid PRF Using Various Centrifugation Protocols

**DOI:** 10.3390/molecules27072043

**Published:** 2022-03-22

**Authors:** Zahra Kargarpour, Layla Panahipour, Richard J. Miron, Reinhard Gruber

**Affiliations:** 1 Department of Oral Biology, Medical University of Vienna, 1010 Vienna, Austria; zahra.kargarpooresfahani@meduniwien.ac.at (Z.K.); layla.panahipour@meduniwien.ac.at (L.P.); 2 Department of Periodontology, School of Dental Medicine, University of Bern, 3012 Bern, Switzerland; richard.miron@zmk.unibe.ch; 3 The Miron Research Lab, Fort Lauderdale, FL 20250, USA

**Keywords:** platelet-rich fibrin, platelet-poor plasma, buffy coat, fibrinogen, blood clot, sticky bone, blood coagulation

## Abstract

Liquid platelet-rich fibrin (PRF) is produced by fractionation of blood without additives that initiate coagulation. Even though liquid PRF is frequently utilized as a natural source of fibrinogen to prepare sticky bone, the concentration of fibrinogen and the overall amount of “clottable PRF” components have not been evaluated. To this aim, we prepared liquid PRF at 300, 700, and 2000 relative centrifugal force (RCF), for 8 min and quantified the fibrinogen levels by immunoassay. We report here that, independent of the RCF, the fibrinogen concentration is higher in the platelet-poor plasma (PPP) compared to the buffy coat (BC) fraction of liquid PRF and further decreases in the remaining red fraction. We then determined the weight of the clotted PRF fractions before and after removing the serum. The PPP and BC fractions consist of 10.2% and 25.3% clottable matrix suggesting that more than half of the weight of clottable BC is caused by cellular components. Our data provide insights into the distribution of fibrinogen in the different fractions of liquid PRF. These findings suggest that PPP is the main source of clottable fibrinogen, while the BC is more a cell source when it comes to the preparation of sticky bone.

## 1. Introduction

Solid platelet-rich fibrin (PRF) was originally introduced as the coagulated plasma fraction of centrifuged blood enriched with platelets and leucocytes and depleted in red blood cells [1]. The original tubes used for blood collection contain a soluble clot activator becoming entrapped in the solid PRF [2]. Alternatively, rough surface glass tubes serve as a clot activator [3]. Solid PRF is, thus, a consequence of the intrinsic coagulation pathway culminating in the activation of thrombin (factor IIa) intrinsic to blood [4]. Thrombin in turn cleaves the N-terminus of the Aα and Bβ chains in fibrinogen, thereby releasing the fibrinopeptides A and B [5]. The nascent fibrin elements in turn self-assemble into fibrin strands that are further crosslinked with factor XIIIa to form the fibrin mesh [5]. Activated platelets accumulating in the plasma further strengthen the formation and stability of the fibrin clot by their GpIIb/IIIa fibrinogen receptor [5]. The so-formed solid PRF appears yellowish and can be further processed into PRF membranes by squeezing out the noncoagulating liquid serum. PRF membranes are then clinically applied for instance to support chronic wound healing [6], treatment of refractory leg ulcers [7], and keratinized tissue augmentation [6].

Liquid PRF is similarly prepared from centrifuged blood with the plasma fraction being depleted from red blood cells [8]. At higher centrifugation forces, blood is fractionated into a cell-free platelet-poor plasma (PPP) and the so-called buffy coat (BC) layer or concentrated C-PRF (C-PRF) where the platelets and leucocytes accumulate [9]. For preparing liquid PRF, the plastic tubes used for blood collection have no additives and a hydrophobic surface to delay the spontaneous activation of the coagulation cascade. Thus, liquid PRF with its PPP and BC layer maintains the native fibrinogen which is ready to undergo coagulation once thrombin is formed. The intrinsic coagulation cascade can be initiated by the presence of collagen, which is considered a sign of injury for the coagulation cascade [10]. From a clinical perspective, collagen being a major component of autologous and, in particular, demineralized allogenic bone grafts favor the coagulation cascade [11].

Sticky bone takes advantage of this principle, where bone or dentin grafts are mixed with liquid PRF waiting for the coagulation cascade to be initiated [12,13,14]. The grafts, also together with minced solid PRF membranes, are then entombed by the fibrin-rich extracellular matrix of the yellow blood clot that hold the cellular components of the BC rich in platelets and leucocytes [12,13,14]. This conglomerate of bone and dentin grafts, minced solid PRF, and the clottable components of liquid PRF is then transplanted into the defect side, which is typically a site requiring bone augmentation such as implant sites following tooth loss [12,13,14]. Even though this procedure is clinically established, some basic questions remain open, particularly with respect to how the centrifugation protocol affects the fibrinogen distribution of the liquid PRF. There are many protocols for preparing liquid PRF at all centrifugation forces ranging from 60× *g* for 3 min [12,13] to 700× *g* for 8 min [9] or 2000–3000× *g* for 8 min [15]. In the present protocols, the amount of fibrinogen was measured in different fractions of liquid PRF. We show here that PPP and the buffy coat are both rich in fibrinogen but there is a gradient that depends on centrifugation forces.

## 2. Methods

### 2.1. Preparation of Liquid PRF

Liquid PRF was prepared after the approval of the Ethics Committee of the Medical University of Vienna (1644/2018), and volunteers signed informed consent. All experiments were performed in accordance with relevant guidelines and regulations. For preparation of liquid PRF fractions, venous blood was collected at the University Clinic of Dentistry from six healthy volunteers at 23 to 35 years, in 9 mL nonridged (pull cap) plastic tubes (“No Additive”, Greiner Bio-One GmbH, Kremsmünster, Austria), and centrifuged at 300× *g*, 700× *g*, and 2000× *g* for 8 min (Z 306, Hermle, Universal Centrifuge, Wehingen, Germany) with universal swing-out rotors (146 mm at the max). Immediately following centrifugation, blood layers were separated. According to the method proposed by Miron et al., a 900 µL volume from the top layer was sequentially pipetted moving downward. The fractions were collected to perform analysis to accurately determine the amount of fibrinogen following centrifugation with various protocols [16,17]. The fractions were transferred into vials containing 100 µL of trisodium citrate solution (BD Vacutainer, PL67BP, Franklin Lakes, NJ, USA) to avoid blood coagulation. Noteworthy, when fraction 5 was drawn, it was possible to visualize the layer separation between the yellow plasma and red blood corpuscle parts, and it was stored at −20 °C before the in vitro analysis. Then, we evaluated the fibrinogen concentration of all the liquid PRF fractions using the human fibrinogen ELISA kit (Aviva System Biology, San Diego, CA, USA).

### 2.2. Measuring Wet and Dry Weight of Fractions of Liquid PRF Clots

Following centrifugation of blood in nonridged plastic tubes at 2000× *g* for 8 min, the first five fractions (each fraction was 900 µL) were collected in individual tubes, supplemented with 2 U/mL of bovine thrombin (Merck, Darmstadt, Germany) and left for 45 min at room temperature to coagulate. The weight of clotted fractions was measured before and after removing the liquid following pressing the clot once with moderate hand pressure toward a sterile gauze. After removing the serum, we obtained a white clot. This procedure is similar to our previously protocol used to prepare PRF membranes [18,19,20]. The clotted fraction before and after removing the serum by a gauze was used to measure the wet weight and dry weight, respectively. The ratio of dry weight/wet weight (D/W) was calculated to determine the percentage of clottable components including the fibrin-rich matrix and the cellular components.

### 2.3. Statistical Analysis

All experiments were performed four to six times. Each data point is representative of processed PRF individually obtained from a different blood donor in the treatment groups. Statistical analysis was performed with the Friedman test for multiple comparison of all groups using fraction 4. Analyses were performed using Prism v8 (GraphPad Software, La Jolla, CA, USA). Significance was set at *p* < 0.05.

## 3. Results

### 3.1. The Fibrinogen Gradient of Liquid PRF

**Figure 1:**
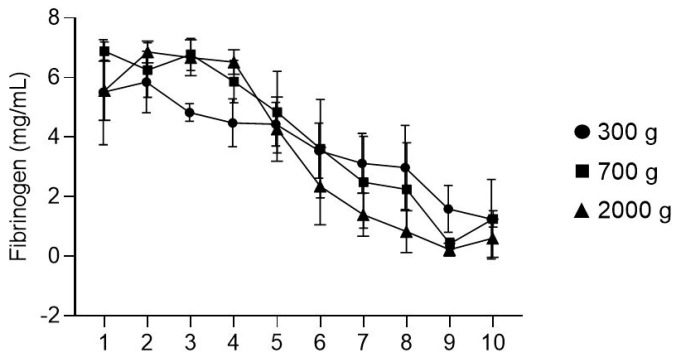
Fibrinogen concentration in 10 fractions of nonclotted centrifuged blood. Liquid PRF was prepared at three different centrifugation speeds, 300× *g*, 700× *g*, and 2000× *g*. As indicated, the fibrinogen concentration decreased after fraction 5, where the so-called BC fraction is located.

To determine the distribution of fibrinogen in liquid PRF, we performed immunoassays with sequential fractions from the top (first) layer down to the last (10th) layer within the PRF tube. At 2000× *g*, the first four fractions representing around 3.6 mL contained a constant concentration of fibrinogen of around 7 mg/mL (Figure 1). There was a sharp decrease in fraction 5 representing the BC with around 4.5 mg/mL fibrinogen. At 700× *g*, the fibrinogen concentration was less constant, but the overall impression was that the decline in the concentration of fibrinogen had already started at fraction 4. Most obvious, at 300× *g*, the distribution showed a steady decline throughout all fractions with no plateau, albeit comparably lower in the typically discarded red fraction. Fibrinogen in the red clot fraction of liquid PRF prepared at 700× and 2000× *g* was similar, reaching around 1 mg/mL in fractions 7 and 8. Thus, 2000× *g* and, to a slightly lesser extent, 700× *g* offered a constant high yield of fibrinogen in the first 3–4 mL of liquid PRF.

### 3.2. The Clottable Fraction of Liquid PRF

**Figure 2:**
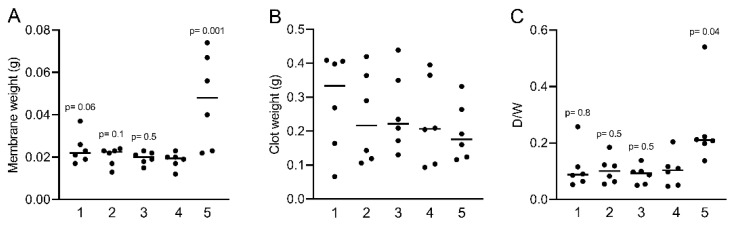
Measuring the clottable fraction of liquid PRF. (**A**,**B**) The weight of clotted liquid PRF prepared at 2000× *g* before and after compressing is indicated. (**C**) The ratio of the pure clotted fibrinogen against total clottable fibrinogen was calculated dry weight/wet weight (D/W). Statistical analysis was based on the Friedman test, and *p*-values are indicated compared to fraction 4 (*N* = 6). Significance was set at *p* < 0.05.

To evaluate to which extent the concentration of fibrinogen in liquid PRF prepared at 2000× *g* translates into a clottable fraction, we measured the weight of the clotted liquid PRF fractions formed. After calculating a ratio of the weight of the wet PRF clot to the respective PRF membrane from which the serum was removed, it emerged that, on average, 10.2% ± 0.06% of the first four fractions of the liquid PRF was clottable (Figure 2A,B). Interestingly, in fraction 5, the relative weight was higher, reaching a mean of 25.3% ± 0.14% (Figure 2C), suggesting that even though the concentration of fibrinogen dropped significantly, the clot became heavier. This was, however, not surprising considering that fraction 5 represents the BC fraction where the platelets and leucocytes accumulate. Importantly, the fibrinogen was sufficient for the coagulation of the BC fraction, suggesting that even the BC fraction with lower fibrinogen levels can contribute to the preparation of sticky bone.

## 4. Discussion

Sticky bone is not a scientific term but commonly used jargon when it comes to the preparation of a conglomerate consisting of bone grafts, minced solid PRF, and the clottable fraction of liquid PRF [14]. The PRF bone block is more scientific in this respect [12,13]. Stickiness comes from the fibrinogen in the liquid PRF that coagulates following the activation of the intrinsic coagulation pathway, which includes not only the collagen provided by the bone graft but also the thrombin released by the solid PRF membranes. Considering that the concentration of fibrinogen may change depending on the RCF and follow a gradient, the present study was performed to investigate this issue. We show here that indeed the concentration flows along a gradient that depends on the RFC, highlighting that 700 and particularly 2000 RCF are ideal for the preparation of around 3–4 mL of clottable liquid PRF, along with a cell-rich fraction within the buffy coat. This research further highlights that the red cell zone is poor in fibrinogen, thereby underlining the possible advantage of preparing liquid PRF rather than using fully unfractionated blood for preparing sticky bone.

If we relate the findings to those of others, we have to acknowledge the research done to date on measuring fibrinogen in the fraction of anticoagulated blood [21]. In the clinical routine of coagulation and hemostasis research, fibrinogen concentration and its clottable activity are major parameters to predict the risk of thrombosis, stroke, heat track, and atherosclerosis [22]. The concept of determining the amount of fibrinogen in translational medicine is also established when it comes to the manufacturing of a fibrin glue used for tissue sealing in filling needle holes after cardiothoracic and vascular surgery [23]. In this case, fibrinogen is enriched by fractionation and cooling to reach a supra-physiological concentration, albeit with the drawback of an allogenic source, the need for virus inactivation and other processing requirements, as well as the additional costs. Autologous fibrinogen is a suitable alternative to allogeneic fibrin glue to prepare sticky bone.

The clinical consequences of the data we present here have to be interpreted with care, but the overall conclusion is in support of the common protocols regarding the preparation of sticky bone [13,14], whereby tubes with no additives and a hydrophobic surface are used to delay the spontaneous activation of the intrinsic coagulation cascade. The ideal RCF and the time are maybe a matter of debate; however, there is a general preference for a higher centrifugation speed (not less than 700× *g*) that generates around 4 mL of liquid fibrinogen enriched with BC cells. What we have not evaluated is how the concentration of fibrinogen in the PPP and BC affects the mechanical and biological properties of the sticky bone. However, if we consider that the amount of clottable fibrin-rich matrix is limited by the amount of fibrinogen in the liquid PRF, the amount of the stickiness can be calculated. For instance, when adding 8 mL of liquid PRF prepared at 2000× *g* to 0.5 g of bone grafts and two minced PRF membranes, the sticky part will consist of around 60 mg of fibrinogen and 150–200 mg of clottable liquid PRF. When considering the fibrinogen simply as a biological glue, the handling properties may dictate the ideal protocol. If it turns out that the fibrin-rich clottable matrix has a biological activity that supports the overall process of graft consolidation, targeting the ideal concentration of fibrinogen and the BC components may become relevant to refine the protocols for the preparation of sticky bone.

The clinical relevance of our work exceeds the original research question related to the preparation of sticky bone as liquid PRF can serve as a source of fibrinogen to prepare PRF membranes simply by waiting until the natural coagulation is initiated, which takes around half an hour without additives. It is relevant, however, to determine how much fibrinogen is in each fraction and the yield of clottable components present to predict the ability of liquid PRF to generate PRF membranes that are presumably easier to handle than standard PRF where the time between vein puncture and centrifugation is a critical determinant of the size of solid PRF [24]. These liquid PRF-derived membranes may be prepared in larger quantities and even stored in a cooling environment until their use, which may be more ideal for the treatment of diabetic wounds [25]. PRF membranes can be customized in shape into larger-sized membranes by pooling of PPP and BC from various tubes. Furthermore, a cryo-precipitation of autologous fibrinogen to increase the overall concentration of the clottable matrix is possible, albeit not easily applicable at present in a dental office setting [26].

Considering that this was a pilot study, there are limitations to acknowledge. Firstly, we enrolled only four to six probands in the study that were all considered as young and healthy with no bleeding disorders or patients taking anticoagulant therapies. Thus, the study does not present the variation of patients that may benefit from the use of liquid PRF to prepare sticky bone. Secondly, we only applied one centrifugation time of 8 min that is clinically applicable, but we did not cover the wide spectrum of possible protocols that include short centrifugation times of as little as 3 min [27]. Thirdly, there are more methods available to measure the concentration and particular rate of clotting of the fibrinogen such as other immunological fibrinogen assays including radial immunodiffusion and immunonephelometric [21] or clotting rate assays [28]. Fourthly, our method to determine the relative clottable fraction of liquid PRF is based on weight measurements and limited to the preparation at 2000× *g*/8 min and not considering protocols of higher/lower RCF with shorter/longer centrifugation times. Lastly, we can only speculate why the gradient develops. We clearly see that less fibrinogen exists in low-speed centrifugation at 300× *g* compared to the 700× *g* and 2000× *g* protocols where the PPP is certainly platelet- and leucocyte-poor. Knowing that the low-speed protocol resulted in a more evenly distributed platelet layer throughout the PRF layers, where the cells are not accumulated in the buffy coat zone, we assume that the cells in the low-speed PPP dilute the fibrinogen [17]. Fibrinogen itself presumably does not generate a gradient; hence, the gradient of fibrinogen in liquid PRF reflects the presence of cellular components. This is even more obvious in the fractions containing erythrocytes. The impact of how cells dilute the fibrinogen in liquid PRF remains at the level of a hypothesis. Nevertheless, our study should be considered as a primer for future discoveries taking all the open questions into account and further refining the overall concept to optimize the use of liquid PRF in the preparation of sticky bone.

## Data Availability

The original contributions presented in the study are included in the article. Further inquiries can be directed to the corresponding author.
